# Modeling of the native knee with kinematic data derived from experiments using the VIVO™ joint simulator: a feasibility study

**DOI:** 10.1186/s12938-024-01279-z

**Published:** 2024-08-23

**Authors:** Paul Henke, Johanna Meier, Leo Ruehrmund, Saskia A. Brendle, Sven Krueger, Thomas M. Grupp, Christoph Lutter, Christoph Woernle, Rainer Bader, Maeruan Kebbach

**Affiliations:** 1 Department of Orthopaedics, Rostock University Medical Center, Doberaner Straße 142, 18057 Rostock, Germany; 2 Research and Development, Aesculap AG, Am Aesculap-Platz, 8532 Tuttlingen, Germany; 3 Department of Orthopaedic and Trauma Surgery, Musculoskeletal University Center Munich (MUM), Campus Grosshadern, Ludwig Maximilians University, Munich, Germany; 4 Chair of Technical Mechanics, University of Rostock, Justus-Von-Liebig-Weg 6, 18059 Rostock, Germany

**Keywords:** Human knee joint, Musculoskeletal modeling, Joint simulator, Cadaveric testing, Ligament resection

## Abstract

**Background:**

Despite advances in total knee arthroplasty, many patients are still unsatisfied with the functional outcome. Multibody simulations enable a more efficient exploration of independent variables compared to experimental studies. However, to what extent numerical models can fully reproduce knee joint kinematics is still unclear. Hence, models must be validated with different test scenarios before being applied to biomechanical questions.

**Methods:**

In our feasibility study, we analyzed a human knee specimen on a six degree of freedom joint simulator, applying a passive flexion and different laxity tests with sequential states of ligament resection while recording the joint kinematics. Simultaneously, we generated a subject-specific multibody model of the native tibiofemoral joint considering ligaments and contact between articulating cartilage surfaces.

**Results:**

Our experimental data on the sequential states of ligament resection aligned well with the literature. The model-based knee joint kinematics during passive flexion showed good agreement with the experiment, with root-mean-square errors of less than 1.61 mm for translations and 2.1° for knee joint rotations. During laxity tests, the experiment measured up to 8 mm of anteroposterior laxity, while the numerical model allowed less than 3 mm.

**Conclusion:**

Although the multibody model showed good agreement to the experimental kinematics during passive flexion, the validation showed that ligament parameters used in this feasibility study are too stiff to replicate experimental laxity tests correctly. Hence, more precise subject-specific ligament parameters have to be identified in the future through model optimization.

**Supplementary Information:**

The online version contains supplementary material available at 10.1186/s12938-024-01279-z.

## Background

Despite tremendous advances in total knee arthroplasty (TKA), only 80%–85% of patients are satisfied with the postoperative outcome [1, 2]. Biomechanical studies are conducted to better understand knee joint kinematics before and after TKA [3–5]. Such studies are performed experimentally or numerically, while computer-based studies need experimental data to be validated [6–8].

On the other hand, studies on patients enable the investigation of the native knee joint and functionality of TKA, considering the soft tissue structures [9]. However, extensive parameter studies are not possible due to ethical aspects. In addition, investigating different motion sequences is technically challenging, and the measurement of kinematics on the patient is partially prone to errors [10]. Contrarily, experimental setups with human specimens allow for examining the knee joint at different complexity scales. For this purpose, mechanical knee rigs [11, 12] and industrial robots [13–15] have been used. Although these setups provide valuable insights into the knee joint dynamics, test rigs usually cannot apply defined loads and torques about all three spatial axes, and the usage of robots can be very complex and time-consuming. To overcome these limitations, the six degrees of freedom joint simulator VIVO™ (Advanced Mechanical Technology, Inc., Watertown, USA) is used to study the kinematics of the native human knee joint and the contribution of specific ligaments to overall joint stability [16, 17]. It can also be used for testing implant components with virtual ligaments represented by numerical strain-force laws [18, 19].

Based on the experimental data, multibody simulations are used to noninvasively analyze the knee joint kinematics and serve as a tool for virtual tests of different implant designs [20–23]. However, the quality of the simulation results highly depends on the boundary conditions used, and it is still unclear to what extent they fully reproduce the kinematics of the human knee joint [24]. Especially the parameters used to model the ligaments are described with a broad range in the literature and significantly contribute to joint kinematics [25].

Therefore, the aim of our present study is to generate a subject-specific multibody model of the right knee joint that mimics an experimental setup for investigating the human knee joint kinematics. For this setup, a knee specimen is experimentally examined on the VIVO™ joint simulator using different load cases, i.e., passive knee flexion and various laxity tests, to characterize knee joint kinematics. Additionally, tests are repeated with a sequential resection strategy of the knee ligaments. Based on medical images of the human specimen, a complex subject-specific multibody model comprising ligament structures and contact between articulating cartilage surfaces is generated to mimic the experimental tests. A more physiological simulation of the human knee joint kinematics will significantly contribute to several biomechanical questions, such as soft tissue management during TKA [26].

## Results

### Experimentally derived knee kinematics

#### Passive flexion

**Fig. 1:**
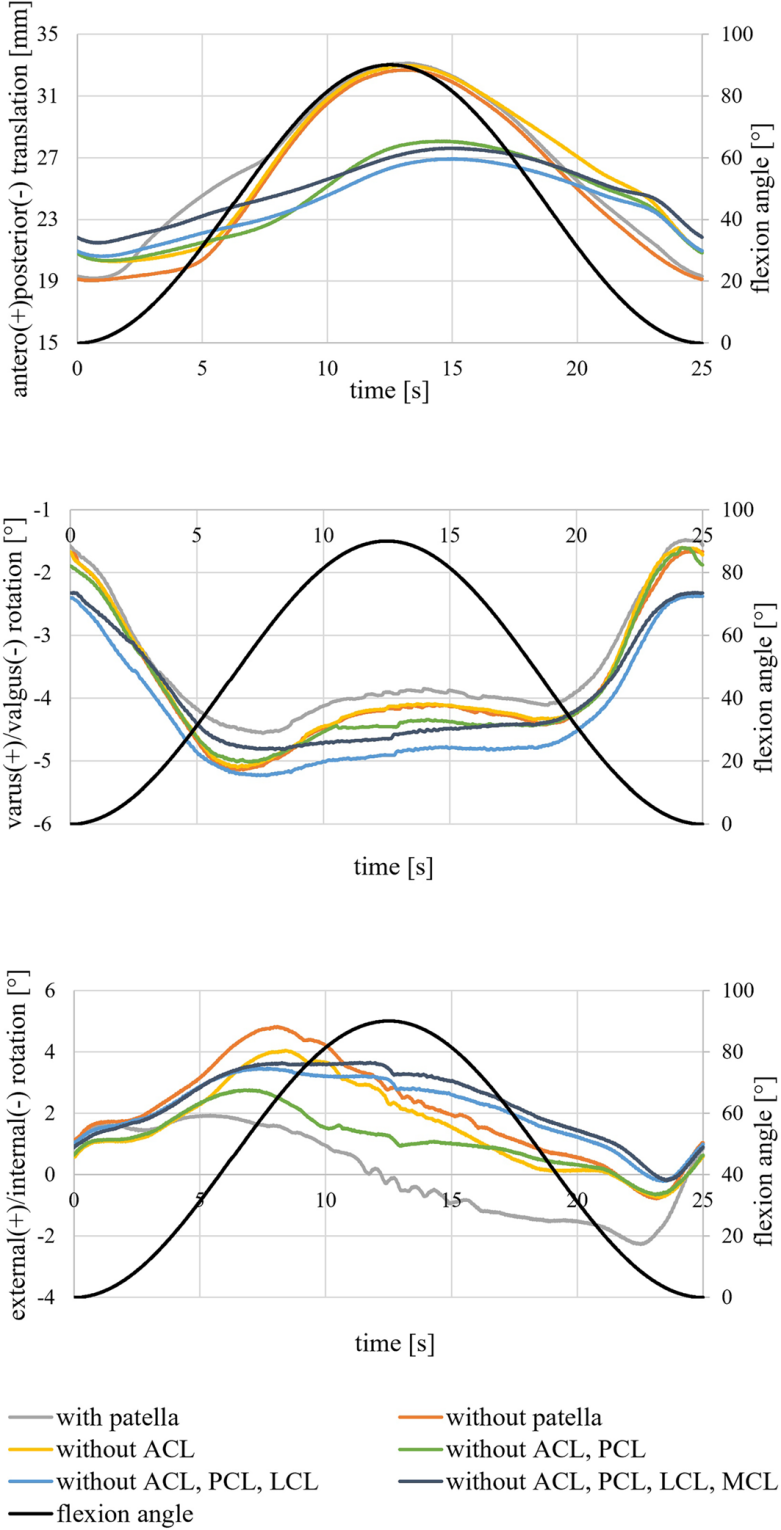
Results of the anteroposterior translation, the varus/valgus rotation, and the external/internal rotation for different resection states during passive flexion and extension. The experimental data shows the position of the tibia coordinate system with respect to the femur coordinate system in Grood and Suntay convention [27]

Figure 1 shows the kinematics over time during the passive knee flexion for the different resection states of the ligaments. Results were evaluated for the tibia coordinate system with respect to the femur coordinate system according to the Grood and Suntay convention [27].

Starting from initial values of 19–22 mm, the anterior translations for all resection states peaked approximately near the highest flexion value at 12.5 s with maximum values between 27 and 33 mm before returning to the original initial values with the shift back to extension. Resection of the patella slightly reduced anterior translation during the transition from flexion to extension, whereas the reverse was unaffected.

The varus/valgus curve showed a similar trend for all resection stages. Starting from a slight valgus position between − 2.4° and − 1.5°, the varus angle constantly decreased until midflexion (30–45° flexion) before it rose to its original value. While resection of the patella and lateral collateral ligament (LCL) decreased the varus/valgus rotation, removal of the medial collateral ligament (MCL) increased the rotation by up to 0.5°.

The course of external/internal rotation was similar for all resection states. It started at the same point at about 1° external rotation, then climbed to a maximum during midflexion before returning to its initial value. The rotation depends on whether it goes into or comes out of flexion. Patellar resection reduced external rotation by up to 3°. Resection of the anterior cruciate ligament (ACL) and especially the posterior cruciate ligament (PCL) further reduced the range of external/internal rotation.

#### Laxity tests

**Fig. 2:**
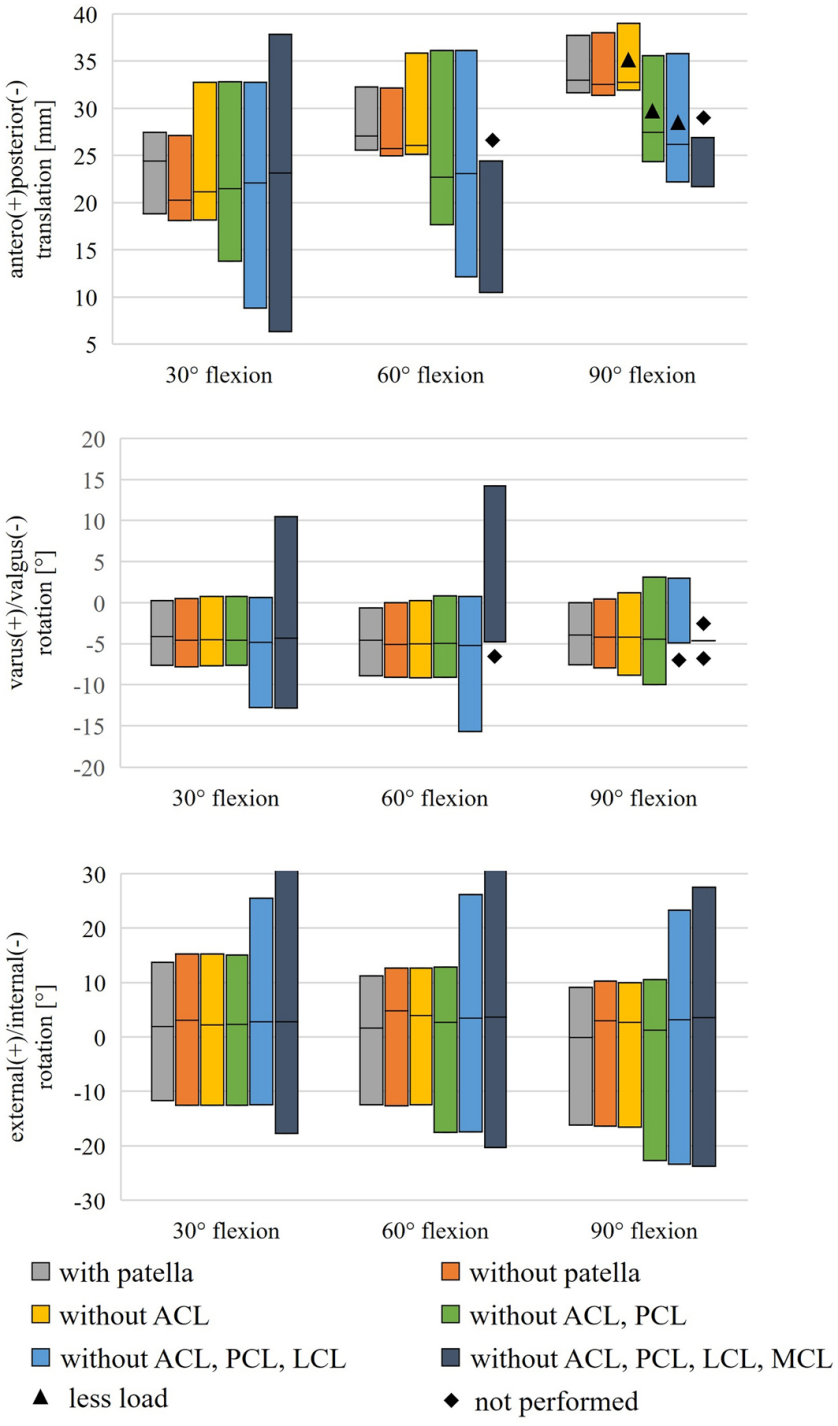
Results of the experimental laxity tests, where the upper end of the bars represents the position of the specimen for 40 N anterior/5 Nm varus/2.5 Nm external load. The lower end represents the position for 40 N posterior/5 Nm valgus/2.5 Nm internal load. The position during passive flexion is marked in between. The loads that had to be reduced to 10 N instead of 40 N due to the limitation of the working space of the joint simulator are marked with a triangle. The trials marked with a rhombus could not be carried out for the same reason

All three translational and rotational degrees of freedom during loading in anteroposterior, varus/valgus, and external/internal directions were recorded over 10 s and averaged to evaluate the laxity tests. The range of movement is shown via bars in Fig. 2. The upper end of the bars represents the position for 40 N posterior/5 Nm varus/2.5 Nm internal loading. A line highlights the position during passive flexion, while the lower end of the bars represents the position for 40 N anterior/5 Nm valgus/2.5 Nm external loading. Resection of the patella had no considerable influence on the knee laxity results. With resection of the ACL, laxity in the anterior direction increased by up to 5 mm for flexion angles of 30° and 60°. At 90° flexion, the load had to be reduced to 10 N due to the limitation of the working space of the joint simulator. These cases are marked with a triangle in Fig. 2. Laxity during anterior loading was not changed by the resection of the PCL and LCL at 30° and 60° flexion. At 90° flexion, the maximum load could not be applied during the working space's limitation. Laxity during posterior loading increased by approximately 5 mm after resecting the PCL, 5 mm after resecting the LCL, and 2.5 mm after resection of the MCL at a flexion angle of 30°. The varus/valgus rotation limits at 5 Nm load showed almost no influence on the patella, ACL, and PCL resection for 30° and 60° flexion angles. However, the resection of the LCL and MCL had a distinct influence on valgus/varus rotation. Some load cases could not be included due to the specimen's instability and are marked with a rhombus in Fig. 2. External and internal rotation limits were not reasonably changed by the patella, ACL, and PCL resection at a 30° flexion angle. For 60° and 90° flexion angles, resecting the PCL increased the internal laxity by about 5°. Resection of the LCL resulted in higher external rotation for all flexion angles, whereas resecting the MCL led to higher external and internal rotation at all flexion angles.

### Numerically derived kinematics of passive flexion

The root mean square error (RMSE) was calculated for the kinematic parameters obtained from the multibody simulations of the passive flexion cycle to compare the numerical data with the experimental results. In the case of translational kinematics, the RMSE was 1.32 mm in the mediolateral direction, 1.61 mm in the anteroposterior direction, and 1.34 mm in the superoinferior direction. For the rotational kinematics, the RMSE was 0.84° for varus/valgus and 2.12° for external/internal rotation.

**Fig. 3:**
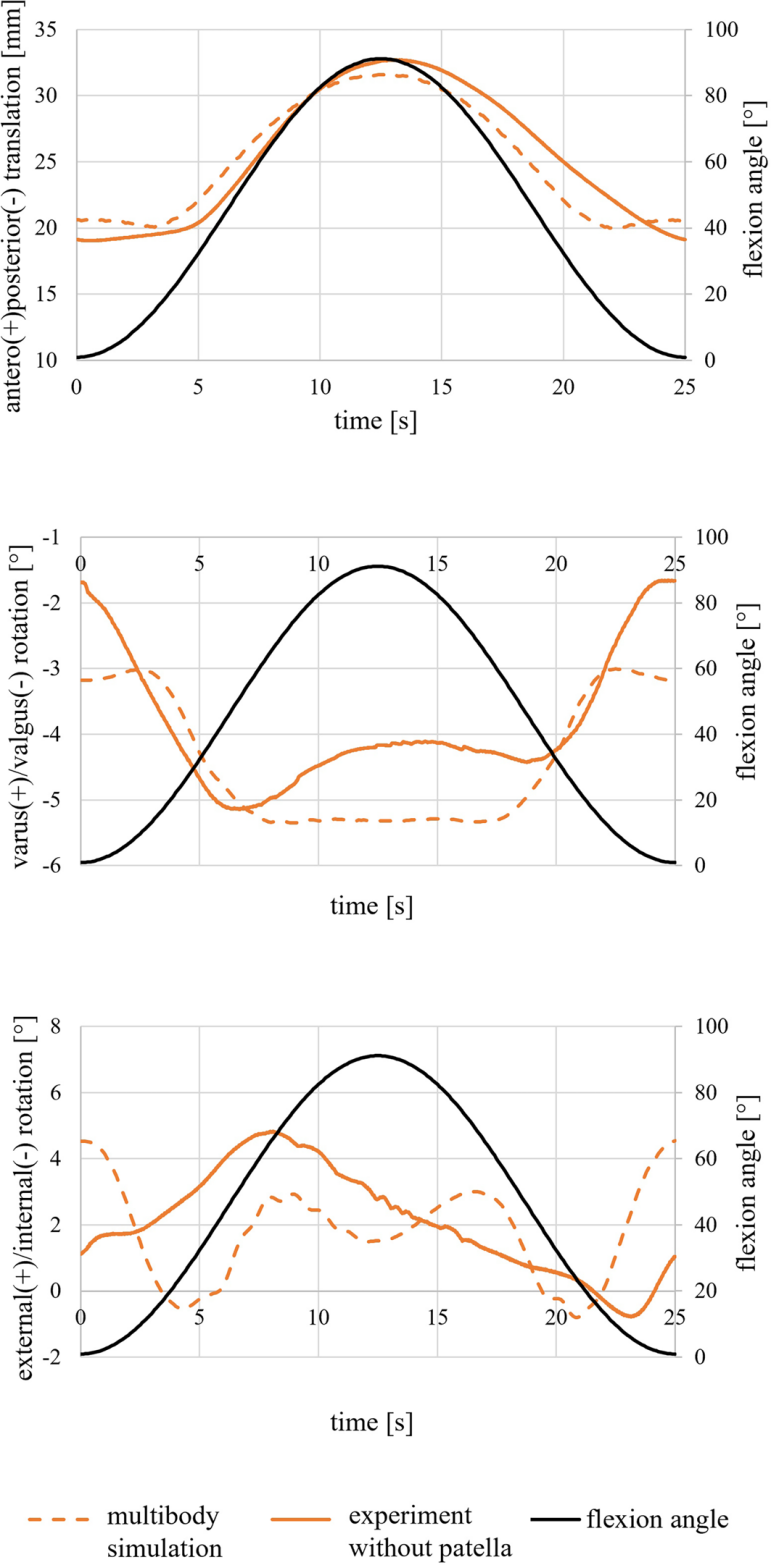
Numerically derived data of the anteroposterior translation, the varus/valgus rotation, and the external/internal rotation compared to experimental results during passive flexion and extension. The experimental results refer to the resection state without the patella but with all ligaments. Data show the position of the tibia coordinate system with respect to the femur coordinate system in Grood and Suntay convention [27]

The data of anterior translation of the tibia with increasing flexion angles derived from the multibody modeling agreed with the experimental kinematics (Fig. 3). The varus/valgus rotation displayed the same progression with increasing flexion angle but slightly different start values and positions during 90° of flexion. For external and internal rotation, numerical and experimental kinematics showed the maximum external rotation at 60° flexion and an external rotation when reaching 90° extension and the end of the motion. The amplitude of the rotations was in the same range, whereby the numerical kinematics showed more deviations in the rotation angle.

**Fig. 4:**
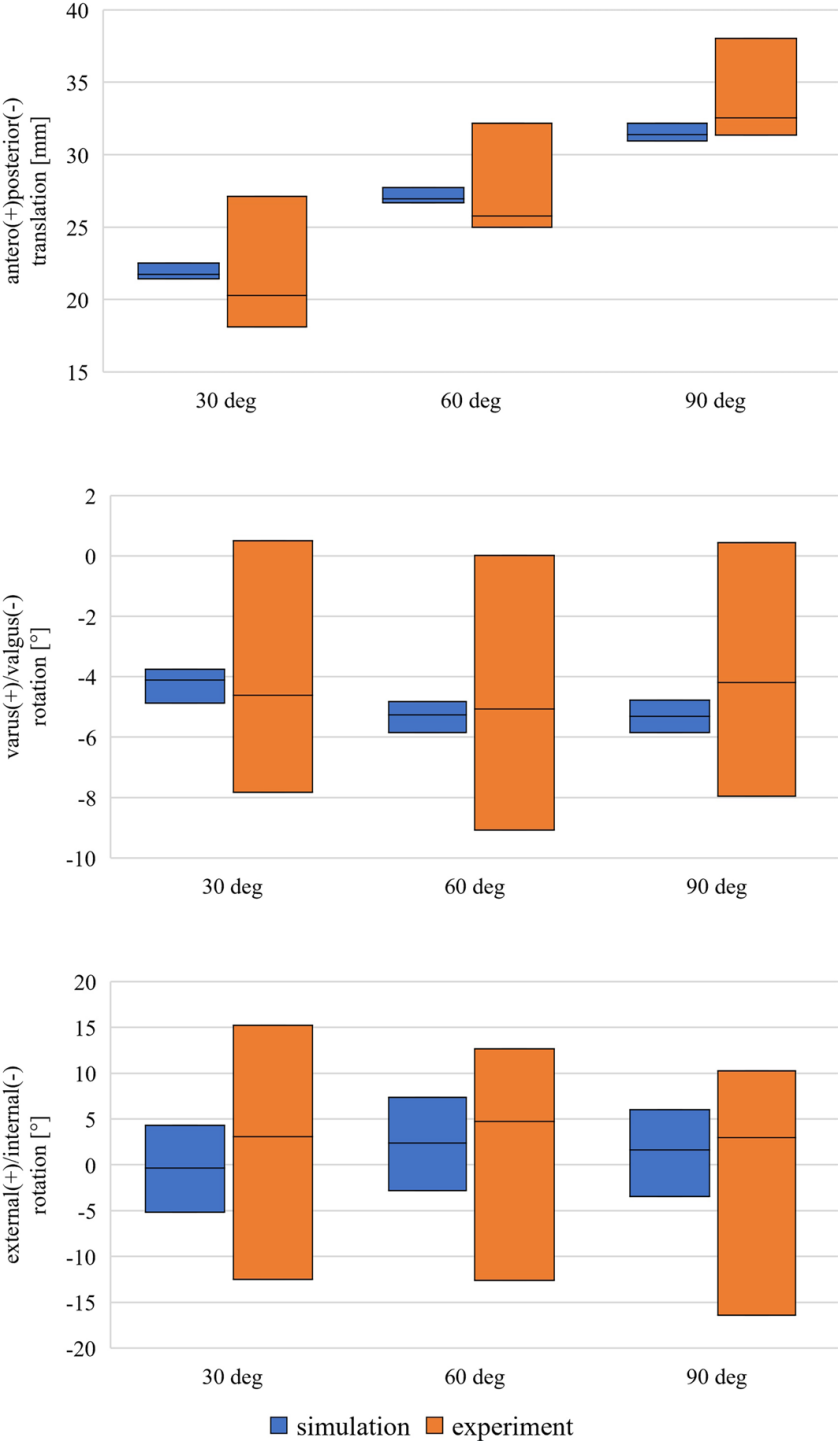
Results of the simulated laxity tests with all ligaments intact. The upper end of the bars represents the position of the specimen for 40 N anterior/5 Nm varus/2.5 Nm external load. The lower end represents the position for 40 N posterior/5 Nm valgus/2.5 Nm internal load. The position during passive flexion is marked in between

A maximum contact force during passive flexion of 1000 N was calculated from the multibody model at a flexion angle of 90°. In general, numerical modeling resulted in lower laxity of the ligaments (Fig. 4). While the experiments showed a range of laxity between 6 and 8 mm in anteroposterior direction for different flexion angles when all ligaments were intact, the numerical model only allowed 2–3 mm of movement when applying the same loads. For varus/valgus laxity, the experiments showed a laxity of about 10°, while in the numerical model, the laxity was reduced to approximately 1.5° for all flexion angles. Laxity for external and internal rotation was also reduced in the numerical model from approximately 25° in the experiments to approximately 10° in the multibody simulation. The influence of sequential resection could not be further investigated in the numerical model, as the resection of individual ligaments led to significant instability.

**Fig. 5:**
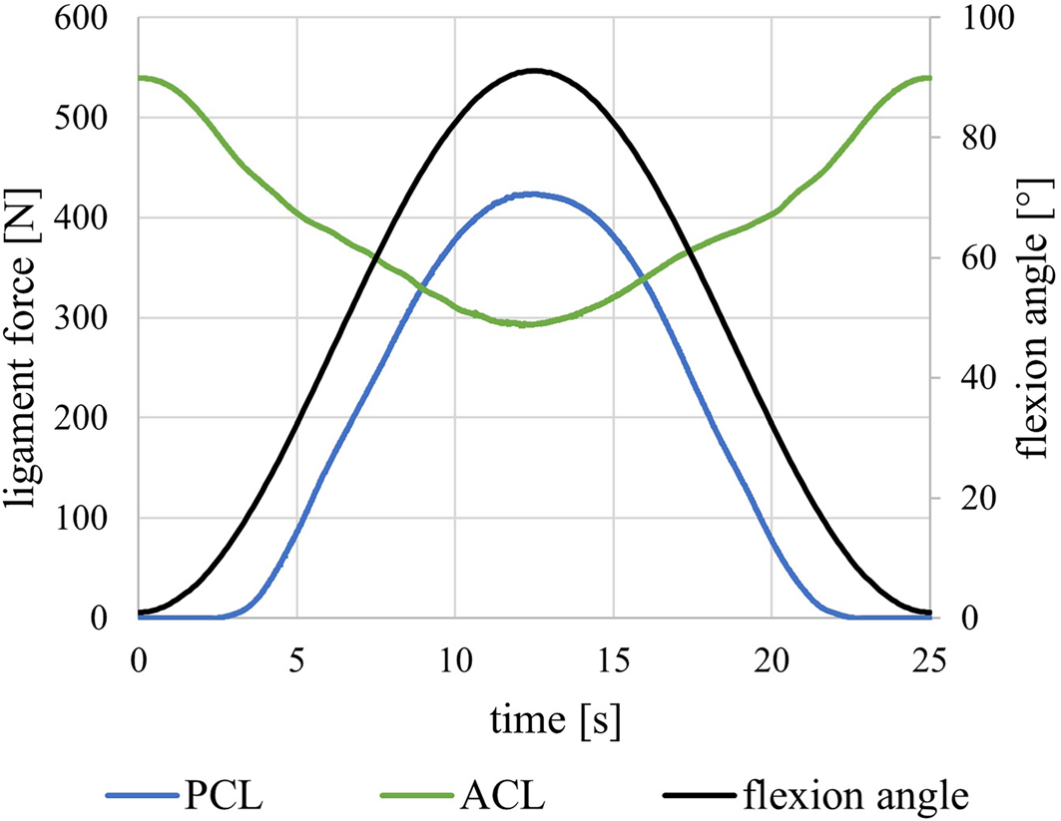
The calculated ACL and PCL ligament forces derived from the multibody model for the passive flexion over time

Ligament forces calculated by the numerical model varied between 300 and 550 N during passive flexion for the ACL and between 0 and 420 N for the PCL. The progression of the forces in the cruciate ligaments is shown in Fig. 5. The force in the ACL decreased with flexion angle, while the PCL force increased with flexion angle. Results for the LCL and MCL forces are available as Additional file 1 in the Supplementary Information.

## Discussion

This feasibility study aimed to investigate individual knee kinematics during different loading scenarios by generating a subject-specific multibody model that mimics the experimental data. The measured kinematics during passive flexion and different laxity measurements were in good agreement with the literature for both native and resected states. Nevertheless, it has to be noted that the direct comparison with other studies is only of limited value due to the different and often insufficiently documented positioning of the joint coordinate systems. In future work the coordinate systems should be defined as standardized as possible (e.g. using 'REFRAME' [28]) to ensure better comparability with studies of other groups.

While the kinematics of passive flexion of the experimental testing could be well reproduced with the multibody model, it showed a reduced range of laxity compared to the experimental tests. We conclude that the ligament parameters from the literature used in our study are too stiff to fully represent our experimental test results.

The test results considering tensile loading of the quadriceps femoris tendon showed increased anterior translation of the tibia during passive flexion. It has been demonstrated in a previous study that isolated muscle loading affects tibiofemoral kinematics [29]. Passive flexion induced an anterior displacement of the tibia, primarily caused by the tension of the PCL with increasing knee flexion [30]. This displacement could be seen in the experimentally recorded data and was comparable to data from other groups [25, 29, 31]. After PCL resection, the translation was less consistent with the literature [25, 32]. In contrast, removing the ACL increased anterior translation of the tibia, especially in early flexion.

The patella or the ligaments' resection did not considerably affect the varus/valgus rotation during passive flexion. There was a slight increase in valgus rotation after the resection of the LCL and a slight rise in varus rotation after the resection of the MCL, corresponding to the ligaments' physiological directions of action. The resections of the collateral ligaments resulted in increased valgus and varus rotation, as both the MCL [33] and the LCL [34] function as stabilisers for varus/valgus rotations in the human knee joint. This agrees with the literature, where the varus/valgus rotation does not depend on the flexion angle [35].

External and internal rotation showed the screw-home mechanism when reaching extension in experimental and numerical data, which is well documented in the literature [25, 31, 32, 36]. When full flexion of 90° was achieved, an internal rotation of approximately 4° was also evident in the experimental results. During the last 10° of extension, a final rotation of approximately 2° could be observed. Resection of the ACL and PCL reduced the changes in external/internal rotation angle during flexion due to missing wrapping of the cruciate ligaments. In general, rotation was more internally rotated after the resection of the ACL, which can be explained physiologically by the lack of wrapping of the cruciate ligaments around each other. Similar observations were documented by Markolf et al. [31]. Resection of the collateral ligaments led to a further reduction in tibial rotation during passive flexion, as the tibial torque was reduced since at least one of the ligaments is usually under tension throughout flexion movements [37].

Liu-Barba et al. [38] described a correlation between the applied compression force and the resulting translations and rotations. Different amounts of applied compression force complicate the comparability between the studies. Increased internal rotation during extension compared with flexion has been similarly documented by Markolf et al. [31]. Testing with the patella and loaded quadriceps femoris tendon also showed increased internal rotation, which may be explained by the individually acting lever arms of the muscle forces and cannot be evaluated with sufficient accuracy without considering a force effect in the hamstring muscles.

Considering the resection states, the results of the laxity tests agreed with the literature data [8]. An almost identical AP laxity during the tests with and without the patella supports the assumption that the differences in kinematics with and without the patella during passive flexion were due to the load applied to the tendon of the quadriceps femoris muscle. Nevertheless, further studies should be performed to verify this assumption. Resection of the ACL increased the possible translation in the anterior direction, and resection of the PCL showed a more posterior translation of the tibia as supported by Willinger et al. [39] and Kennedy et al. [40]. In addition, the possible internal rotation during laxity tests after resection of the ACL and PCL was higher, especially at large flexion angles, which is plausible since the wrapping of the cruciate ligaments no longer restricts internal rotation. Resection of the LCL essentially allowed more valgus rotation, while removal of the MCL increased varus rotation. In addition, more external rotation was possible after the resection of the LCL and more rotation in general after the resection of the MCL, which can explain the instability of the knee joint. The translation values during anterior and posterior loading of the intact preparation were lower than those of Moslemian et al. [36]. However, they agreed well with the data of Pedersen et al. [8]. On the other hand, the values of varus/valgus and external/internal rotations were comparable to the results of Moslemian et al. [36].

Nevertheless, the comparison is limited because the applied loads and the definition of the coordinate systems may differ in several studies. More precisely, the loads applied in this study were reduced by 50% compared to those documented in previous literature [8, 16, 17]. This modification was necessary as initial trials with increased loads exceeded the machine's working space after multiple resections due to rising laxity.

A multibody model of the tibiofemoral joint has been created to mimic the experimental kinematics during passive flexion and laxity tests. The RMSE was considerably low for all degrees of freedom. Hence, a good agreement was reached between experimental and numerical kinematics. The range of motion of the tibial anteroposterior translation in the simulation was slightly lower compared to the experiment. This could be due to stiffer cruciate ligaments in the multibody model restricting the range of motion.

The varus/valgus curve also showed a similar curve in the multibody simulation. However, it started with about 1.5° more valgus rotation. Contrary to the experimentally measured data, whether the flexion angle was approached from flexion or extension was also not essential in the multibody model, as the values do not differ. This is a general difference in comparing the kinematics between the experiment and the multibody model, which becomes even more apparent when considering the external/internal rotation. While the numerical curves show a symmetry axis in the middle of the load cycle (at 12.5 s), the experiment's results differ between the extension and flexion phases. This may be due to the neglected time dependency of the ligament properties. The reduced range of motion in rotation in comparison to the literature was also reflected by the model. The resection of a single ligament led to a considerable imbalance in the simulation, which the remaining ligaments could not compensate for.

While the same progression of change in the ACL and PCL force over passive flexion was shown in other models [25, 41–43] and experimental studies [43], the value of the forces in the simulation is considerably higher. The decreased range of movement in simulated laxity tests indicates that ligament forces in the model are much higher than in the experiment. Too high ligament stiffness is probably the reason for model instabilities in the simulation of the individual resection stages. This points out the necessity for further optimization of ligament modeling.

Some limitations of our feasibility study have to be taken into consideration. First, only one human specimen was tested, which does not provide a statistical indication of the reproducibility of the tests. This specimen was preconditioned before the start and rewetted at regular intervals between the experiments to ensure the same test conditions before each test. Nevertheless, it can not be excluded that the physiology changed over the testing period of eight hours. The friction properties of the contact surfaces on the preparation may also have changed over time. Physiological lubrication conditions were assumed in the tests on the intact specimen. After patellar resection, the contact surfaces were continuously wetted with sodium chloride to prevent them from drying out. However, this may also have an influence on the friction properties of the preparation due to different properties compared to human synovial fluid [44]. In the experimental setup, when testing the intact joint, only the quadriceps muscles were loaded over the patella and only with a constant force, while the posterior muscles were neglected. When performing the tests, only the joint reaction forces were controlled. However, we do not have any information about the actual contact forces acting in the joint, which would have been beneficial for the subsequent validation of the multibody model.

Regarding the multibody model, limitations were mainly associated with ligament modeling and tibiofemoral contact. While being three-dimensional organic structures, ligaments were modeled as one-dimensional force spring elements. However, according to the literature [37], this assumption is legitimate to simulate the ligaments accurately. The multibody model does not use time-dependent force elements for the ligaments, whereas in-vivo, this property can be attributed to the viscoelasticity of the ligaments and the cartilage [45]. As this may be a reason for differences in the symmetry of the kinematic, the influence of time dependence when modeling the ligaments should be investigated in the future. Wrapping the ligament around bones or other soft tissues was omitted. In future work, the virtual ligament model of the joint simulator shall be used, which does not provide such wrapping around obstacles [46–48]. In addition, previous studies [37, 49] showed that the absence of wrapping is essential for non-physiological loading and movement scenarios, which were not investigated in this work. Moreover, identifying suitable ligament parameters is still challenging as the reported parameters in the literature are inconsistent [37]. The results of the numerical laxity tests indicate that the entire ligament apparatus is slightly too stiff to reproduce the experimental investigations more accurately. The computation of ligament force as Wismans' method, which does not consider viscoelasticity, is also predefined by the virtual ligament modeling used in the joint simulator. This factor could explain the observed asymmetry in the experimental studies, which was not replicated numerically. The current model is also limited by the contact implementation, where the menisci are neglected, and viscoelasticity is missing at the contact element between the femoral and tibial cartilage. In addition, fluid effects, which have an influence on contact mechanics in numerical studies, are not considered by the contact element used [50, 51]. Furthermore, the parametrization of contact modeling is always a challenge in biomechanical simulations [37].

## Conclusions

In our feasibility study, a human fresh-frozen specimen was successfully investigated experimentally on a complex six-degree-of-freedom joint simulator with passive flexion and different laxity tests under sequential ligament resections. A subject-specific multibody model of the tibiofemoral joint was generated and showed good agreement with the experimental kinematics during passive flexion. Differences in the laxity tests between the multibody model and the experimental investigations suggest that ligament parameterization is still challenging and needs further improvement. Even with a good kinematic agreement between experiment and simulation for the passive flexion, it is necessary to validate multibody models with different load cases that especially consider the stiffness of specific ligaments, such as laxity tests. Currently, a multibody optimization algorithm is developed to determine subject-specific ligament parameters using the experimental results obtained in this work. By coupling the multibody model with an optimisation algorithm, the ligament parameters will be iterated until the error between the experimentally determined kinematics and the calculated model kinematics is minimized [14]. These ligament parameters can be used in future experimental studies using the joint simulator and in computational investigations of knee joint dynamics under different loading scenarios.

## Methods

### Experimental testing

**Fig. 6:**
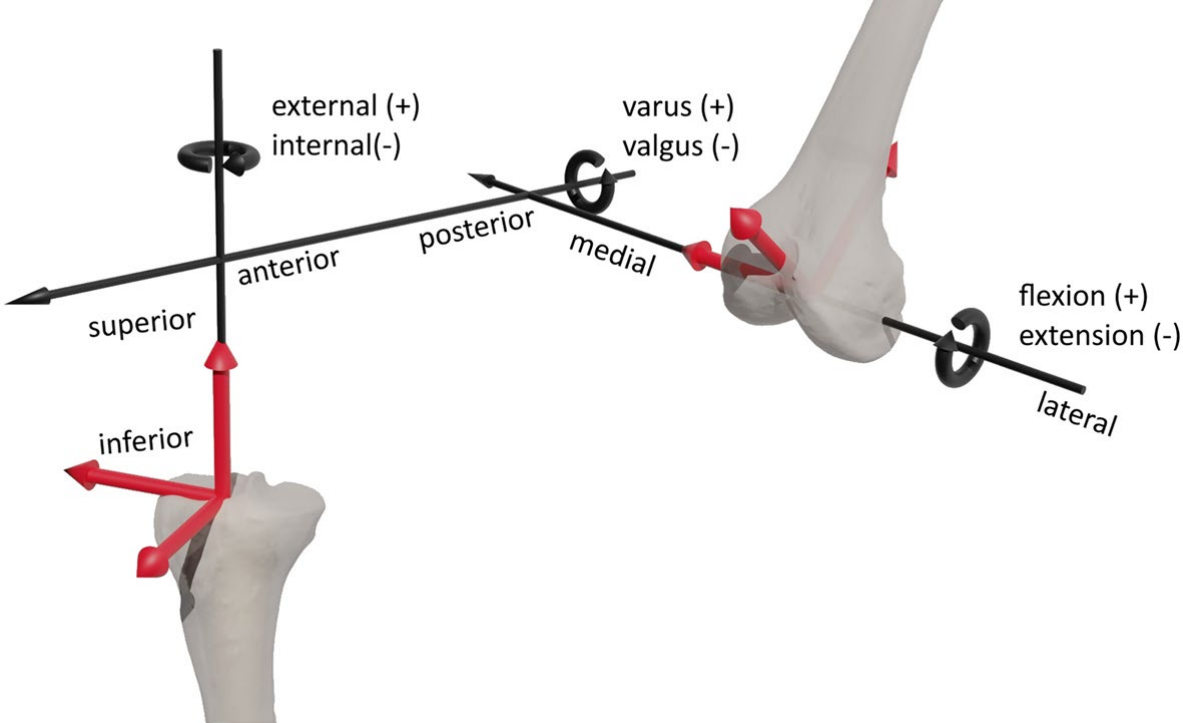
Depiction of the femoral and tibial joint coordinate systems, with the flexion/extension axis fixed to the femur coordinate system, the external/internal rotation axis fixed to the tibia coordinate system and the varus/valgus rotation axis defined as a floating axis perpendicular to these two according to Grood and Suntay [27]

Ethical approval was obtained from the Institutional Review Board (A 2023-0055) for our experimental testing. Before preceding the experiments on the VIVO™ joint simulator, a fresh-frozen human specimen (male, 76 years, 56.7 kg) of the right knee was analyzed by computed tomography (SOMATOM Perspective, Siemens Healthineers, Erlangen, Germany). Based on these data, bone structures of the fibula, tibia, femur as well as the femoral cartilage structures were segmented and reconstructed with the software Amira 5.4.1 (Thermo Fisher Scientific, Waltham, Massachusetts, USA and Zuse Institute Berlin, Berlin, Germany). The tibial cartilage was measured and digitised with an Artec Space Spider 3D scanner (Artec 3D, Senningerberg, Luxembourg), as it was not possible to clearly distinguish between tibial cartilage and menisci during the segmentation process using the CT scan of the frozen specimen. All cartilage surfaces were processed in Geomagic Studio 2013 (3D Systems, Rock Hill, South Carolina, USA). Joint coordinate systems were defined according to the literature [52–54]. Relative motion between the femur and tibia was defined in Grood and Suntay coordinates [27]. In Fig. 6, a brief overview of the convention of Grood and Suntay is depicted based on [27].

**Fig. 7:**
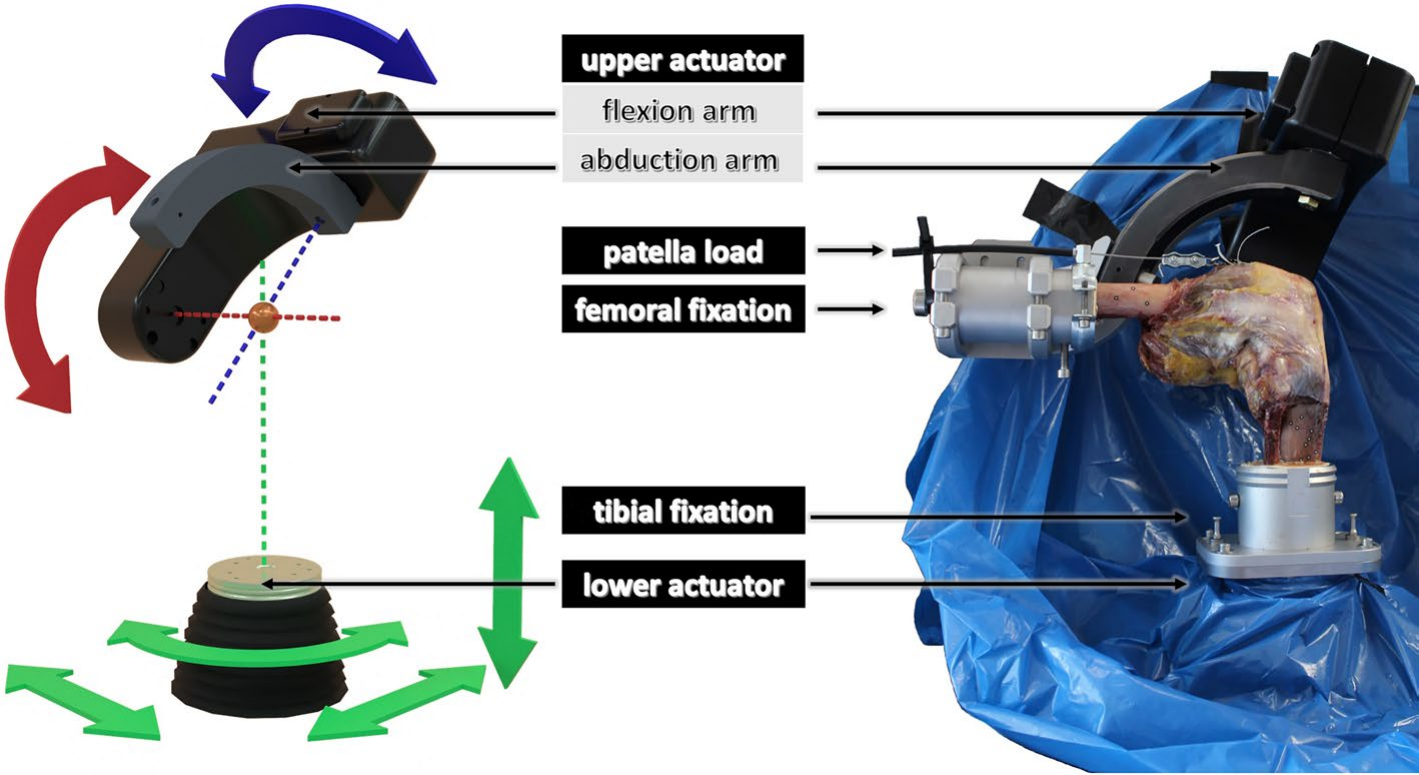
Illustration of the various degrees of freedom of the VIVO™ simulator (left) with the upper actuator enabling two rotations, one with the flexion arm (red) and one with the abduction arm (blue). The lower actuator has one rotational and three translational degrees of freedom (green). The experimental setup (right) has the femur and tibia attached to the upper and lower actuators of the VIVO™ joint simulator. The constant tensile load was applied to the patella via a cable

Two actuators realize the movement of the joint simulator. The upper actuator consists of an abduction arm coupled with a flexion arm, thus enabling two rotations (flexion/extension, varus/valgus-rotation). The lower actuator, the xyz table, can perform three translations and one rotation around the vertical axis (internal/external). During the test, a kinematic loop equation maps the movements of the machine's hydraulic actuators to the Grood and Suntay coordinates in real time [55]. The knee was aligned in the joint simulator to match the previously defined femoral and tibial joint coordinate systems. Thus, the knee joint flexion axis corresponded to the joint simulator's flexion axis. For fixation, the femoral and tibial bones were resected proximally and distally and embedded in fast-curing resin GP 010 (Gößl und Pfaff, Brautlach, Germany) to ensure the correct positioning (Fig. 7). VIVO^TM^s degrees of freedom, as well as the experimental setup, are depicted in Fig. 7.

Regarding the experimental protocol, the human specimen was thawed for twelve hours before testing. Afterward, the specimen was moved with 20 cycles of passive flexion to precondition the soft tissues [55]. Different resection states were performed, and simultaneous knee joint kinematics were recorded during the passive flexion and laxity tests. Before each recording, the specimen was moved with at least two cycles of passive flexion for preconditioning purposes. To simulate passive flexion, the flexion axis of the simulator was position-controlled with a prescribed flexion angle as a sine wave with a period 25 s for a cycle between full extension (0°) and flexion (90°). The two other rotational degrees of freedom, the varus/valgus rotation and the tibial internal/external rotation, were torque-controlled to a constant value of 0 Nm.

In comparison, the three translation axes were force-controlled to 0 N for the two horizontal axes and to 50 N for the vertical axis representing a compressive load. The same compressive load was applied for the laxity tests, while flexion angles of 30°, 60° and 90° were prescribed. Precisely, loads in anterior/posterior, varus/valgus, and external/internal directions were applied for 10 s, according to Table 1. At the same time, forces and torques of all other degrees of freedom were force- and torque-controlled to 0 N and 0 Nm, respectively. The data were generated with 100 Hz frequency and smoothed using a four-pole Butterworth filter. The applied loads were adapted from the literature [8, 16, 17].

**Table 1:** Overview of the load cases with applied flexion angles and forces/torques for passive flexion, anterior–posterior (AP), varus/valgus (VV) and internal–external rotation (IE) laxity test

Load case	Flexion (+)/extension (−)	Varus (+)/valgus (−)	External (+)/internal (−) Rotation	Medial (+)/lateral (−)	Anterior (+)/posterior (−)	Superior (+)/inferior (−)
Passive Flexion	0–90° (sine)	0 Nm	0 Nm	0 N	0 N	− 50 N
AP-Laxity	30°, 60°, 90°	0 Nm	0 Nm	0 N	±40 N	− 50 N
VV-Laxity	30°, 60°, 90°	±5 Nm	0 Nm	0 N	0 N	− 50 N
IE-Laxity	30°, 60°, 90°	0 Nm	±2.5 Nm	0 N	0 N	− 50 N

**Fig. 8:**
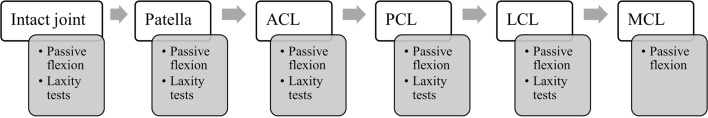
Protocol for the sequential resection states during the experiment with passive flexion and laxity tests performed. Knee joint kinematics was measured at every resection state (except where the joint became too unstable or exceeded the machine's workspace)

For all tests of the knee specimen before the resection states, the patella was loaded via the quadriceps tendon with a constant tensile force of 20 N to stabilize the patella during testing [56]. The first resection state included resecting the patella and, thus, the quadriceps load. After repeated testing, including passive flexion and laxity tests according to Table 1, further resection states include the anterior cruciate ligament (ACL), the posterior cruciate ligament (PCL), the lateral collateral ligament (LCL) and the medial collateral ligament (MCL) (Fig. 8).

### Multibody model

The multibody model of the tibiofemoral joint was generated in the software Simpack (v2022, Dassault Systèmes, Vélizy-Villacoublay, France) to mimic the previously described experimental setup. The reconstructions of the CT dataset were used to set up a multibody topology with the femur and tibia. The multibody simulation did not include the patellofemoral joint, as the additional contact considerably increases the complexity. The femoral and tibial coordinate systems were implemented according to the experiment.

Regarding the multibody topology, the femur was fixed in the world coordinate system. A five degrees of freedom joint with contact between femoral and tibial cartilage was defined to connect the tibia to the femur so that the flexion could be specified at any time. The five degrees of freedom were set according to their state of equilibrium depending on internal (contact) and external (ligaments) forces. Ligaments were modeled as non-linear spring force elements according to Wismans et al. [57], and Blankevoort et al. [58], with the exerting force defined by

1 $$f = \left\{ {\begin{array}{*{20}c} 0 \\ \frac{1}{4}{{\frac{{k\varepsilon ^{2} }}{{\varepsilon _{l} }}}}\\ {k\left( {\varepsilon - \varepsilon _{l} } \right)} \\ \end{array} } \right.\begin{array}{*{20}c} {\varepsilon < 0} \\ {0 \le \varepsilon \le 2\varepsilon _{l} } \\ {\varepsilon > 2\varepsilon _{l} } \\ \end{array} ,$$

where $\varepsilon$ is the strain, $k$ the stiffness and ${\varepsilon }_{l}$ a constant set to 0.03 [59]. The strain can be calculated with

2 $$\varepsilon = \frac{{l - l_{0} }}{{l_{0} }},$$

where *l* is the actual length of the ligament and $l_{0}$ is the slack length of the ligament. Accordingly, the slack length can be calculated from the reference length $l_{r}$ and the reference strain $\varepsilon_{r} ,$

3 $$l_{0} = \frac{{l_{r} }}{{\varepsilon_{r} + 1}}.$$

Regarding the multibody model, the ACL and PCL, the LCL, and the MCL are divided into a superficial and deep medial collateral ligament (sMCL, dMCL) and the oblique popliteal ligament (OPL) consisting of two bundles each (anterior and posterior bundle). Additionally, one bundle each was added for the posterior oblique ligament (POL), the arcuate popliteal ligament (APL), and medial and lateral posterior capsular structures (mCAP, lCAP). Ligament insertion points were identified using the CT data, literature [60–63] and bony landmarks and were approved by an experienced board-certified orthopedic surgeon. The mechanical parameters of the ligaments used in the simulation are based on a previous study [20] and are summarized in Table 2.

**Table 2:** Ligament parameters for stiffness k, reference strain ${\varepsilon }_{r}$ and reference length ${l}_{r}$ used in the multibody simulation for this study

Ligament	Stiffness $k$ [N]	Reference strain ${\varepsilon }_{r}$ [%]	Reference length ${l}_{r}$ [mm]
ACLa	1000	10	31.8
ACLp	1000	3	22.9
PCLa	3000	− 10	32.4
PCLp	1500	− 3	31.1
LCLa	2250	− 25	74.4
LCLp	2250	8	74.5
sMCLa	4000	4	93.4
sMCLp	1500	4	94.4
dMCLa	1500	2	29.7
dMCLp	2000	-7	32.8
OPLa	1250	6	83.2
OPLp	1250	6	85.5
mCAP	2500	5	58.9
lCAP	2500	5	60.9
POL	2000	5	39.5
APL	1500	4	80.8

**Fig. 9:**
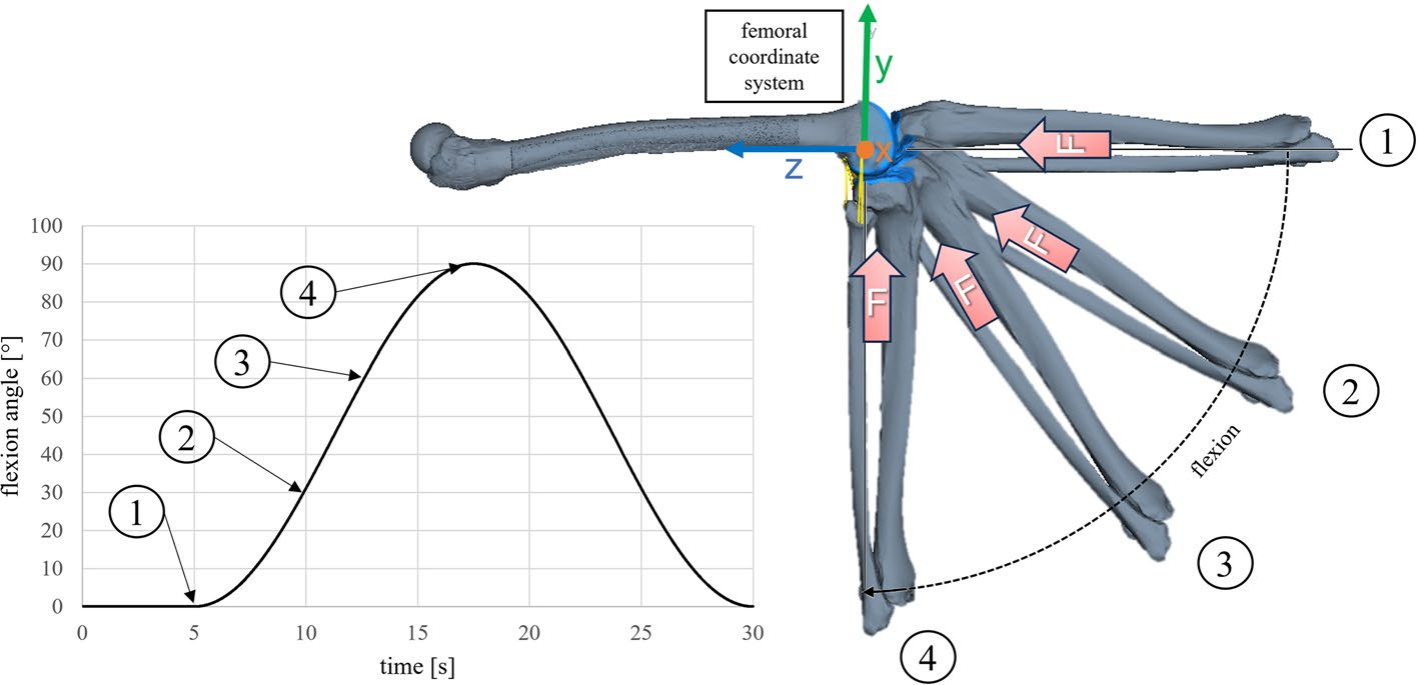
Multibody model of the right knee joint after patella resection during different phases of passive flexion for 0°, 30°, 60°, and 90° with ligaments in yellow and contact surfaces in blue. A five degree-of-freedom rheonomic joint forces a rotation of the tibia around the femoral flexion axis (x-axis). In addition to contact and ligament force elements, a constant compressive force (F) of 50 N was applied

To consider the interaction of the articulating surfaces, a contact force element between femoral and tibial cartilage was defined using an elastic foundation model according to Hippmann et al. [64]. The compressive force of 50 N in the experiment and the loads required for the laxity tests were applied via external force elements in the multibody model. Gravity was neglected to mimic the experimental setup. Figure 9 shows the multibody model of the right knee with the prescribed flexion angle for passive flexion.

### Supplementary Information

**Additional file 1.** Predicted LCL and MCL forces of the multibody model.

## Data Availability

The datasets used and/or analysed during the current study are available from the corresponding author on reasonable request.

## List of symbols

$l$: Actual ligament length
${l}_{0}$: Slack length of the ligament
${l}_{r}$: Reference length of the ligament
$$k$$: Ligament stiffness
$\varepsilon$: Actual strain of the ligament
${\varepsilon }_{l}$: Transition strain—transition from quadratic region to linear region for 2*ε*_*l*_
$${\varepsilon }_{r}$$: Reference strain

## Abbreviations

TKA: Total knee arthroplasty
ACL: Anterior cruciate ligament
PCL: Posterior cruciate ligament
LCL: Lateral collateral ligament
MCL: Medial collateral ligament
sMCL: Superficial medial collateral ligament
dMCL: Deep medial collateral ligament
POL: Posterior oblique ligament
APL: Arcuate popliteal ligament
mCAP: Medial posterior capsular structures
lCAP: Lateral posterior capsular structures
RMSE: Root mean square error
