# Supplementary material for: Modeling of the native knee with kinematic data derived from experiments using the VIVO™ joint simulator: a feasibility study
Source: Biomed Eng Online. 2024 Aug 23;23:85. doi: 10.1186/s12938-024-01279-z (PMC11342559; doi:10.1186/s12938-024-01279-z)
Supplement: Supplementary file 1 — Additional file 1. Predicted LCL and MCL forces of the multibody model. [file 12938_2024_1279_MOESM1_ESM.docx]

**Supplementary material**

1. ***Predicted LCL and MCL forces of the multibody model***


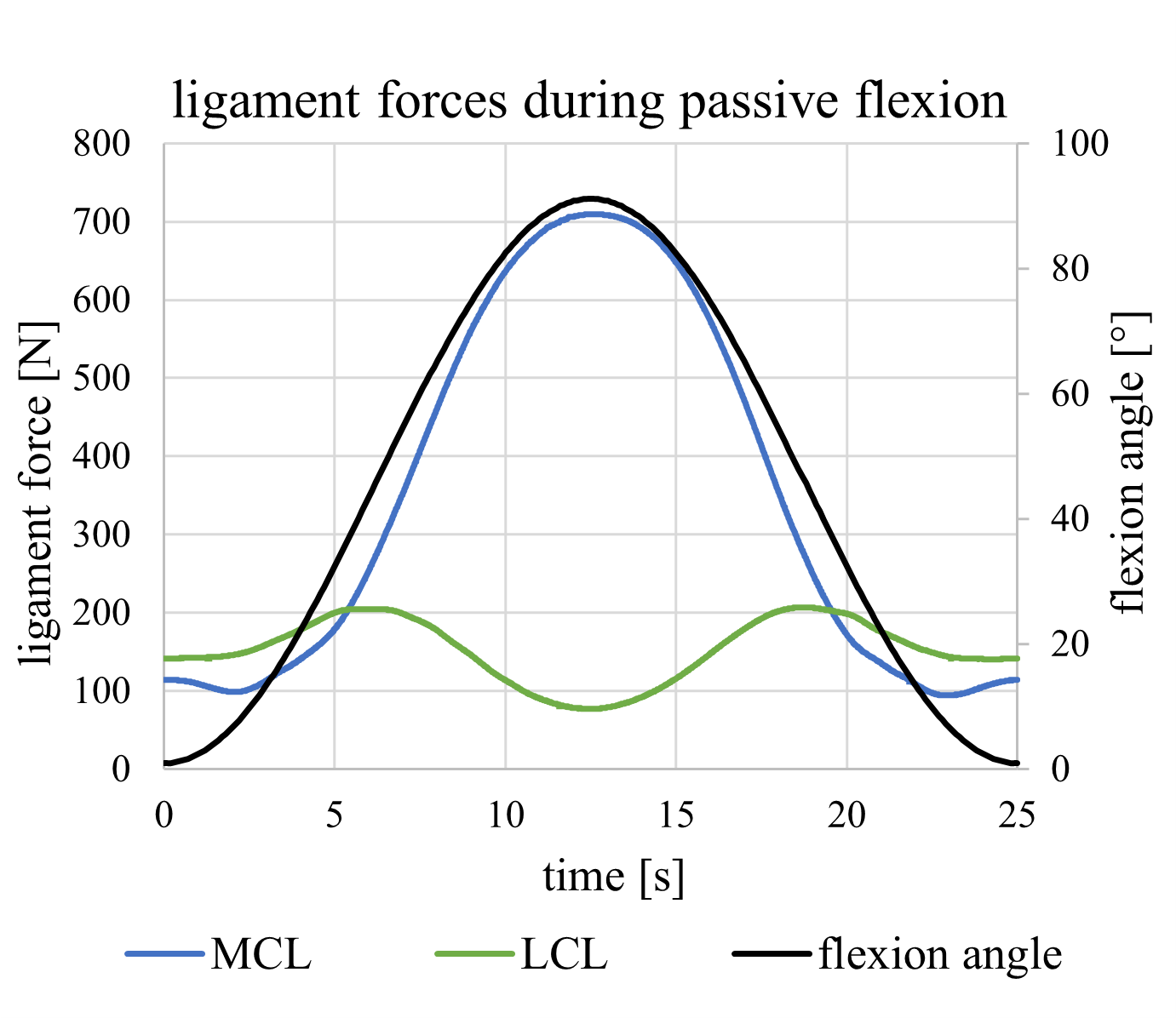
Fig. 1 shows the progression of the forces in the lateral and medial ligaments of the multibody model during passive flexion.

**Figure 1:** **The calculated LCL and MCL ligament forces derived from the multibody model for the passive flexion over time**
